# Hybrid State–Space and Vision Transformer Framework for Fetal Ultrasound Plane Classification in Prenatal Diagnostics

**DOI:** 10.3390/diagnostics15222879

**Published:** 2025-11-13

**Authors:** Sara Tehsin, Hend Alshaya, Wided Bouchelligua, Inzamam Mashood Nasir

**Affiliations:** 1 Faculty of Informatics, Kaunas University of Technology, 51368 Kaunas, Lithuania; sara.tehsin@ktu.edu (S.T.); inzamam.nasir@ktu.edu (I.M.N.); 2 Applied College, Imam Mohammad Ibn Saud Islamic University (IMSIU), Riyadh 11432, Saudi Arabia; hialshaya@imamu.edu.sa

**Keywords:** fetal ultrasound, prenatal diagnostics, state–space models, vision transformers, multi-task learning, 68T07

## Abstract

**Background and Objective**: Accurate classification of standard fetal ultrasound planes is a critical step in prenatal diagnostics, enabling reliable biometric measurements and anomaly detection. Conventional deep learning approaches, particularly convolutional neural networks (CNNs) and transformers, often face challenges such as domain variability, noise artifacts, class imbalance, and poor calibration, which limit their clinical utility. This study proposes a hybrid state–space and vision transformer framework designed to address these limitations by integrating sequential dynamics and global contextual reasoning. **Methods**: The proposed framework comprises five stages: (i) preprocessing for ultrasound harmonization using intensity normalization, anisotropic diffusion filtering, and affine alignment; (ii) hybrid feature encoding with a state–space model (SSM) for sequential dependency modeling and a vision transformer (ViT) for global self-attention; (iii) multi-task learning (MTL) with anatomical regularization leveraging classification, segmentation, and biometric regression objectives; (iv) gated decision fusion for balancing local sequential and global contextual features; and (v) calibration strategies using temperature scaling and entropy regularization to ensure reliable confidence estimation. The framework was comprehensively evaluated on three publicly available datasets: FETAL_PLANES_DB, HC18, and a large-scale fetal head dataset. **Results**: The hybrid framework consistently outperformed baseline CNN, SSM-only, and ViT-only models across all tasks. On FETAL_PLANES_DB, it achieved an accuracy of 95.8%, a macro-F1 of 94.9%, and an ECE of 1.5%. On the Fetal Head dataset, the model achieved 94.1% accuracy and a macro-F1 score of 92.8%, along with superior calibration metrics. For HC18, it achieved a Dice score of 95.7%, an IoU of 91.7%, and a mean absolute error of 2.30 mm for head circumference estimation. Cross-dataset evaluations confirmed the model’s robustness and generalization capability. Ablation studies further demonstrated the critical role of SSM, ViT, fusion gating, and anatomical regularization in achieving optimal performance. **Conclusions**: By combining state–space dynamics and transformer-based global reasoning, the proposed framework delivers accurate, calibrated, and clinically meaningful predictions for fetal ultrasound plane classification and biometric estimation. The results highlight its potential for deployment in real-time prenatal screening and diagnostic systems.

## 1. Introduction

Fetal ultrasound is one of the most widely used imaging modalities for prenatal diagnostics due to its non-invasive, safe, and cost-effective nature. Accurate identification of standard fetal ultrasound planes is crucial for obtaining reliable biometric measurements, detecting anomalies, and making informed clinical decisions. Standard views, such as the head, abdomen, femur, thorax, and spine, provide critical information about fetal growth and development. However, the classification of these planes remains challenging due to significant intra- and inter-subject variability, speckle noise, operator dependency, and differences in acquisition protocols across hospitals and ultrasound devices [1,2].

Traditional machine learning and convolutional neural network (CNN)-based models have achieved considerable success in fetal ultrasound analysis. CNNs are well-suited for extracting local texture and structural patterns, and models such as SonoNet demonstrated the potential of deep architectures for real-time detection of standard planes [1]. Nevertheless, CNN-based methods often suffer from limited receptive fields, making them less effective at capturing long-range dependencies and contextual anatomical relationships. Furthermore, their performance is sensitive to class imbalance and domain shifts, which are common in clinical practice [3,4].

Recent advances in vision transformers (ViTs) have demonstrated that self-attention mechanisms can capture global contextual information across entire images, resulting in remarkable performance in medical image classification and segmentation tasks [5,6]. In the domain of fetal ultrasound, transformers have been explored for standard plane detection and head circumference estimation, highlighting their ability to model distant anatomical dependencies [5]. However, transformers typically require large datasets for stable training and may suffer from poor calibration and overfitting when applied to smaller, noisy medical datasets.

Complementary to transformers, state–space models (SSMs) have recently emerged as powerful sequence modeling tools, offering linear computational complexity and stable modeling of long-range dependencies. Unlike recurrent neural networks, SSMs can efficiently capture temporal or sequential relationships without suffering from the vanishing gradient problem. When applied to medical imaging, SSMs have the potential to model repetitive textures, sequential dependencies across image patches, and global structural continuity, making them a suitable candidate for fetal ultrasound analysis [7,8].

Despite these advances, existing approaches face several limitations that restrict their clinical adoption. First, most methods focus solely on classification accuracy, while ignoring clinically relevant factors such as calibration and interpretability. Second, noisy ultrasound characteristics such as speckle and shadowing remain difficult to address, particularly when training data is limited. Third, robust generalization across datasets acquired under different clinical conditions is rarely validated, which is essential for real-world deployment [9,10]. Therefore, there is a strong need for frameworks that integrate local, sequential, and global modeling capabilities, while ensuring interpretability, robustness, and calibration.

To address these gaps, this work proposes a hybrid state–space and vision transformer framework for fetal ultrasound plane classification and biometric estimation. The architecture integrates the sequential modeling capability of SSMs with the global contextual reasoning of ViTs, enabling a synergistic combination of local anatomical fidelity and global structural coherence. The framework incorporates a preprocessing pipeline for ultrasound harmonization, multi-task learning (MTL) with anatomical regularization, and gated fusion for balancing feature contributions. Furthermore, confidence calibration is explicitly optimized using temperature scaling and entropy regularization to provide reliable predictions for clinical decision-making.

The key contributions of this study can be summarized as follows:

- We introduce a hybrid state–space and vision transformer (SSM-ViT) architecture that unifies sequential and spatial feature modeling for robust fetal ultrasound plane classification.
- We propose a gated and residual fusion mechanism that adaptively balances local continuity captured by the SSM and global semantics extracted by the ViT, supported by detailed ablation and sensitivity analyses.
- We integrate a temperature-scaled confidence calibration module to improve predictive reliability, achieving consistent performance across three independent datasets without retraining.
- We provide a comprehensive empirical evaluation on Fetal_Planes_DB, Fetal Head (Large), and HC18, demonstrating superior accuracy, calibration, and cross-domain generalization compared to state-of-the-art baselines.

The remainder of this paper is organized as follows. Section 2 reviews related work on fetal ultrasound plane classification and recent advances in deep learning architectures. Section 3 presents the proposed hybrid state–space and vision transformer framework, including preprocessing, hybrid encoding, multi-task anatomical regularization, and fusion with calibration. Section 4 reports the experimental setup and results, including ablation studies and comparisons with state-of-the-art (SOTA) methods. Section 5 presents a discussion of the findings and Section 6 concludes the study by outlining future research directions.

## 2. Related Work

Automated analysis of fetal ultrasound images has been an active research area due to the critical role of accurate plane classification in prenatal diagnostics. Early approaches were based on handcrafted features combined with classical classifiers, but their performance was limited by sensitivity to noise, operator dependency, and acquisition variability [11,12]. With the advent of deep learning, convolutional neural networks (CNNs) have become the dominant paradigm, enabling the end-to-end learning of discriminative representations. One of the most influential studies was SonoNet, which demonstrated real-time detection of standard scan planes using a CNN backbone [1]. Extensions such as Att-CNN and ACNet have introduced attention mechanisms to highlight discriminative regions, thereby improving classification performance in complex fetal imaging scenarios [2,3]. Despite these advances, CNNs are inherently constrained by their local receptive fields, limiting their ability to capture global spatial dependencies [4].

To overcome these limitations, transformer-based architectures have been increasingly explored. ViTs leverage self-attention to model long-range dependencies across the entire image, achieving SOTA results in several medical imaging tasks [13,14]. In fetal ultrasound, transformer models such as DiffusionViT and ViTUS have shown promise for plane classification and biometric estimation, demonstrating the ability to capture holistic anatomical structures [5,15,16]. However, ViTs often require large-scale training datasets and are prone to overfitting when applied to smaller, noisy medical datasets [6,17].

In parallel, SSMs have emerged as efficient tools for sequence modeling, capable of handling long dependencies with linear complexity. Frameworks such as HiPPO and S4 introduced stable parameterizations for long-sequence modeling, offering advantages in capturing structural continuity [7,8]. Recent work has explored the integration of SSMs into vision backbones, showing improved scalability and expressiveness compared to pure CNN or transformer approaches [18,19]. Their potential for medical imaging, particularly in ultrasound, where sequential textures and anatomical consistency are essential, is increasingly recognized.

Another important line of research involves MTL and anatomical regularization. Studies have combined plane classification with auxiliary tasks such as segmentation or biometric regression to improve generalization and enforce anatomical plausibility [9,10,20]. Such joint optimization acts as an inductive bias, guiding networks toward clinically relevant feature representations. In addition, extensive data augmentation strategies have been employed to mitigate the limited size of medical datasets and improve robustness against acquisition variability [9,21].

Calibration and reliability of predictions remain critical for clinical adoption. Deep networks often generate overconfident outputs, which can mislead clinicians in sensitive diagnostic workflows [22]. To address this, calibration techniques such as temperature scaling and entropy regularization have been proposed, significantly improving the trustworthiness of automated systems in medical applications [23,24].

CNNs established strong baselines for fetal ultrasound analysis but are limited in capturing global dependencies. ViTs address global reasoning but face challenges with data requirements and stability, while SSMs offer efficient long-range modeling with promising scalability. MTL, anatomical regularization, and calibration further enhance robustness and clinical alignment. These complementary trends highlight the need for hybrid architectures that integrate sequential and global reasoning, ensuring robustness and reliability—gaps that this study aims to address.

## 3. Hybrid Framework for Fetal Ultrasound Plane Classification

The proposed framework introduces a hybrid architecture that combines SSMs with ViTs to achieve accurate and generalizable classification of fetal ultrasound standard planes. The overall pipeline is structured into five stages: image preprocessing for noise reduction and harmonization, hybrid feature encoding through sequential and global context modeling, MTL guided by anatomical regularization, decision fusion with confidence calibration, and interpretability for clinical validation.

### 3.1. Image Preprocessing for Ultrasound Harmonization

Fetal ultrasound images are inherently noisy and heterogeneous in appearance due to differences in acquisition devices, scanning protocols, maternal body habitus, and fetal orientation. Before feeding the data into the hybrid encoder, a robust preprocessing pipeline is required to normalize intensity ranges, suppress speckle noise, align anatomical regions, and generate augmented views for improved generalization. These operations were consistently applied to all images from the three selected datasets: FETAL_PLANES_DB, HC18, and the large-scale fetal head dataset. The first step addresses intensity normalization to mitigate domain-specific biases arising from machine settings such as probe gain and depth adjustments. Each image $I\in R^{H\times W}$ is standardized using z-score normalization:

(1) $$I_{n}\left(x,y\right)=\frac{I(x,y)-\mu (I)}{\sigma (I)+\epsilon },\;\left(x,y\right)\in \left[1,H\right]\times \left[1,W\right],$$

where μ(I) and σ(I) denote the global mean and standard deviation, and ϵ is a stabilizer. After this transformation, the processed images satisfy

(2) $$E\left[I_{n}\right]=0,\;\mathrm{Var}\left(I_{n}\right)=1,$$

which harmonizes brightness distributions across datasets and improves training convergence by ensuring balanced feature scales. The second step addresses speckle noise, a characteristic multiplicative artifact in ultrasound imaging caused by echo interference. It can be modeled as

(3) $$I\left(x,y\right)=I_{c}\left(x,y\right)\cdot n\left(x,y\right),$$

where $I_{c}$ represents the clean anatomical signal and n(x,y) follows a Rayleigh or Gamma distribution. To suppress noise while retaining structural boundaries, anisotropic diffusion filtering is applied:

(4) $$I_{t+1}\left(x,y\right)=I_{t}\left(x,y\right)+\lambda \nabla \cdot \left(c\left(x,y,t\right)\nabla I_{t}\left(x,y\right)\right),$$

with conduction coefficient

(5) $$c\left(x,y,t\right)=\exp\left(-\frac{{∥\nabla I_{t}\left(x,y\right)∥}^{2}}{K^{2}}\right).$$

**Figure 1:**
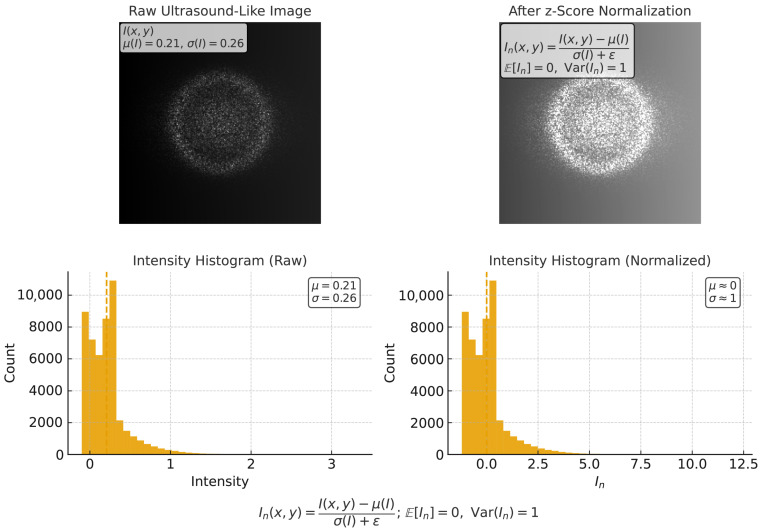
Intensity normalization applied to fetal ultrasound scans to ensure consistent pixel distributions across datasets. This step mitigates inter-subject intensity variability while preserving anatomical structures.

**Figure 2:**
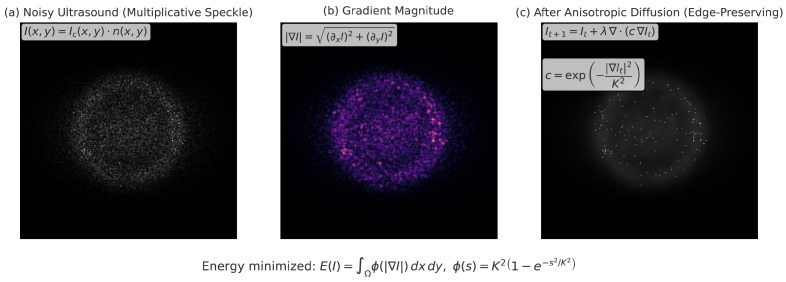
Anisotropic diffusion filtering applied to enhance structural continuity while removing speckle noise from fetal ultrasound images. This step ensures edge preservation for downstream segmentation and attention modules.

This formulation reduces smoothing near edges by modulating diffusion strength based on gradient magnitude. To harmonize the intensity distributions across different subjects and datasets, an intensity normalization step was applied to each fetal ultrasound scan, as visualized in Figure 1. The process minimizes the energy functional.

(6) $$E\left(I\right)=\int_{\Omega }\phi \left(∥\nabla I\left(x,y\right)∥\right)\;dx\;dy,$$

where $\phi \left(s\right)=K^{2}\left(1-\exp\left(-s^{2}/K^{2}\right)\right)$ acts as an edge-preserving penalty, producing enhanced anatomical contrast essential for distinguishing standard planes. The third step ensures geometric consistency through affine alignment, which standardizes resolution and orientation across samples. For each output pixel (x,y) in the aligned image $I_{a}$, its source coordinate $\left(x^{\prime },y^{\prime }\right)$ is determined by

(7) $$\left[\begin{array}{c} x^{\prime }\\ y^{\prime }\\ 1 \end{array}\right]=A\left[\begin{array}{c} x\\ y\\ 1 \end{array}\right],$$

where $A\in R^{3\times 3}$ is defined as

(8) $$A=\left[\begin{array}{ccc} s_{x}\cos\theta & -s_{y}\sin\theta & t_{x}\\ s_{x}\sin\theta & \;\;s_{y}\cos\theta & t_{y}\\ 0 & 0 & 1 \end{array}\right],$$

with $\left(s_{x},s_{y}\right)$ as scaling factors, θ as rotation, and $\left(t_{x},t_{y}\right)$ as translations. To further refine the image quality, anisotropic diffusion filtering was employed to reduce speckle noise while preserving key structural boundaries, as demonstrated in Figure 2. The aligned image is then sampled by bilinear interpolation:

(9) $$I_{a}\left(x,y\right)=I_{n}\left(x^{\prime },y^{\prime }\right),\;\left(x^{\prime },y^{\prime }\right)=\mathrm{bilinear}\left(A{\left[x,y,1\right]}^{T}\right).$$

This guarantees that all images are rescaled to a uniform 224×224 resolution, allowing consistent patch tokenization in the hybrid encoder. The final step enhances robustness through data augmentation, which simulates the variability present in real-world clinical acquisitions. Each aligned image $I_{a}$ is transformed by *T*, sampled from a distribution T of augmentations including random rotations, horizontal flips, and local contrast enhancement via CLAHE:

(10) $$I_{aug}=T\left(I_{a}\right),\;T∼T.$$

**Figure 3:**
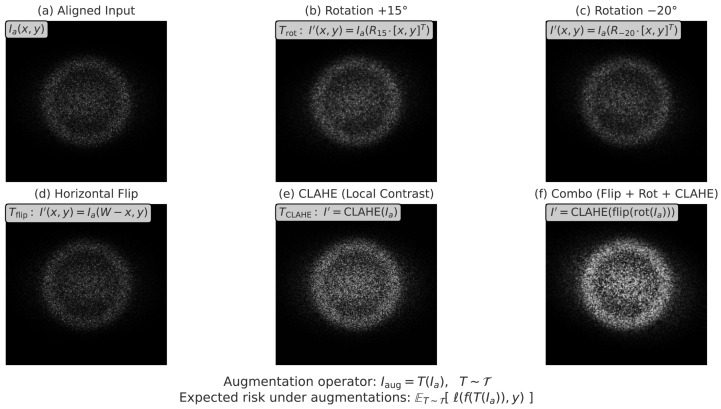
Sample augmentations applied during training, including geometric transformations (rotation, flipping, scaling), intensity jittering, and occlusion simulation. These operations increase robustness to intra-class variability and artifacts inherent in fetal ultrasound.

**Figure 4:**
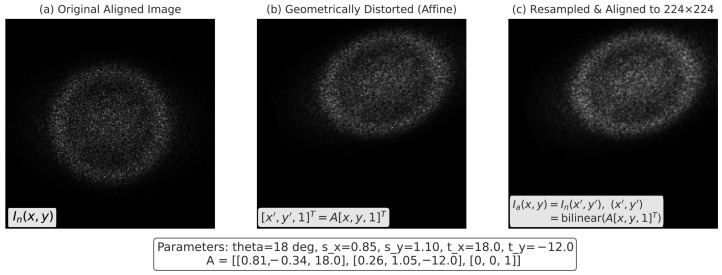
Affine transformation applied to standardize fetal ultrasound views across subjects. This normalization step reduces variability in orientation and scale, facilitating consistent patch embedding and downstream feature extraction.

As shown in Figure 3, diverse augmentation techniques were employed to simulate variability and improve generalization under limited training data. This stochastic process effectively minimizes expected risk across augmented distributions:

(11) $$E_{T∼T}\left[\ell \left(f\left(T\left(I_{a}\right)\right),y\right)\right]\approx \mathrm{RobustRisk}\left(f,I_{a}\right),$$

where *ℓ* is the task loss and *f* the hybrid encoder. To mitigate inter-subject variation, affine alignment was applied to enforce a canonical view across ultrasound samples, as visualized in Figure 4. By averaging risk over transformed views, the model generalizes better to unseen acquisition settings, maternal conditions, and fetal positions, thereby improving reliability in clinical practice.

### 3.2. Patch Embedding and Tokenization for Sequential Representation

Once the ultrasound images are preprocessed and spatially aligned, they are decomposed into smaller, non-overlapping patches to bridge the gap between convolutional feature extraction and sequence modeling. This operation converts the two-dimensional image into a series of discrete tokens that the proposed hybrid state–space and transformer encoder can process. In practice, the aligned image $I_{a}\in R^{H\times W}$ with resolution 224×224 is divided into $N=\frac{H}{p}\times \frac{W}{p}$ patches, each of size p×p. Mathematically, the ith patch is expressed as

(12) $$P_{i}=I_{a}\left[x_{i}:x_{i}+p,\;y_{i}:y_{i}+p\right],\;i\in \left\{1,\ldots ,N\right\},$$

where $\left(x_{i},y_{i}\right)$ represents the top-left coordinate of the patch. Each patch encodes local anatomical details such as fetal skull boundaries, thoracic cavities, or abdominal contours within its receptive field. After partitioning, each patch must be mapped into a continuous feature space suitable for sequence modeling. To achieve this, we apply a linear projection to each flattened patch vector, producing a *d*-dimensional representation. This operation ensures that every patch, regardless of its raw dimensionality, contributes a consistent embedding to the sequence. Formally, the embedding for patch *i* is obtained as

(13) $$z_{i}=W_{p}\cdot \mathrm{vec}\left(P_{i}\right)+b_{p},\;z_{i}\in R^{d},$$

where $W_{p}\in R^{d\times (p^{2})}$ is a learnable weight matrix and $b_{p}\in R^{d}$ is a bias vector. This transformation preserves local structures while reducing input dimensionality and aligning all tokens into a common feature space. The collection of all projected patches forms a structured token sequence, which serves as the direct input to the subsequent hybrid encoder. Specifically, the embeddings are arranged as

(14) $$Z=\left[z_{1},z_{2},\ldots ,z_{N}\right],\;Z\in R^{N\times d}.$$

This formulation treats the image as a one-dimensional sequence of tokens in the same way that words are modeled in natural language processing. In the context of ultrasound, each token acts as a localized “visual word”, representing unique textures or anatomical cues that collectively define the standard plane class. Since transformers are inherently permutation-invariant, it is essential to embed positional information that encodes the spatial order of the patches. Without this step, the network would be unable to differentiate whether a patch corresponds to the fetal brain, abdomen, or femur region in the image. To remedy this, a positional encoding $p_{i}$ is added to each token $z_{i}$, yielding

(15) $$e_{i}=z_{i}+p_{i}.$$

In our framework, positional encodings are treated as learnable vectors rather than fixed sinusoidal functions. This can be expressed as

(16) $$p_{i}=W_{pos}\cdot \phi \left(i\right),$$

where ϕ(i) encodes the two-dimensional patch coordinates $\left(x_{i},y_{i}\right)$ and $W_{pos}$ projects them into $R^{d}$. In this way, the spatial configuration of patches is preserved within the token sequence. To enable image-level classification, a special learnable class token $z_{cls}\in R^{d}$ is prepended to the sequence. After processing by the hybrid encoder, this token aggregates global contextual information from all other tokens. The augmented sequence is thus defined as

(17) $$Z^{\prime }=\left[z_{cls},e_{1},e_{2},\ldots ,e_{N}\right],\;Z^{\prime }\in R^{(N+1)\times d}.$$

The final representation of $z_{cls}$ acts as a compact descriptor summarizing the entire ultrasound image, which is later used for fetal plane classification. To improve training stability and prevent numerical instability, all token embeddings are normalized prior to being passed into the state–space and transformer encoder. This is achieved through layer normalization:

(18) $${\tilde{e}}_{i}=\mathrm{LN}\left(e_{i}\right)=\frac{e_{i}-\mu \left(e_{i}\right)}{\sqrt{\sigma^{2}\left(e_{i}\right)+\epsilon }}⊙\gamma +\beta ,$$

where $\mu (e_{i})$ and $\sigma (e_{i})$ represent the mean and variance across dimensions, while γ and β are learnable scaling parameters. This ensures that all tokens are consistently scaled, which accelerates convergence and enhances representation learning. Collectively, the patch embedding and tokenization stage transforms raw ultrasound images into a structured sequence of tokens, capturing both local anatomy and spatial relationships, thereby laying the foundation for effective hybrid state–space and transformer modeling.

Traditional models combining either LSTMs or CNNs with ViTs are models that combine local feature extraction or temporal recurrence with global transformer modeling. Nevertheless, these operators place some restrictions on ultrasound data. For instance, CNN kernels extract fixed size spatial neighborhoods, but they do not model long range dependencies. Similarly, recurrent LSTM cells suffer from the problem of gradient decay, and regardless, the model’s receptive field is limited when modeling continuous anatomical motion. The SSM defines a continuous-time linear dynamical system in which the state evolves: $h_{t}=Ah_{t-1}+Bx_{t}$, yielding an implicit convolution kernel k=exp(A)B that can be efficiently computed with 1D convolution. This formulation allows for modeling long-range dependencies with linear complexity and stable gradients, as it allows smooth transitions across neighboring anatomy frames. In this manner, the SSM branch facilitates ViT based representations by providing local continuity and dynamic context with respect to motion, while the ViT sense long-range spatial relationships globally.

### 3.3. State–Space Model Encoder for Sequential Dependency Modeling

**Figure 5:**
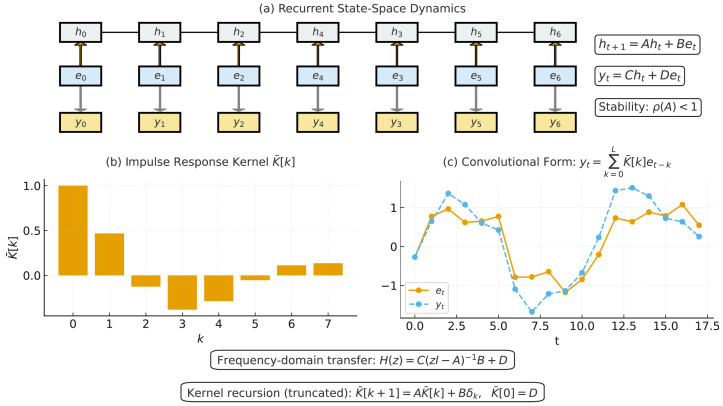
The SSM encoder captures long-range dependencies using recurrent-style dynamics. The input image features are convolved and then passed through a diagonal state transition and an input-dependent observation matrix. This enables structured spatial modeling with low latency.

The patch token sequence produced in the previous stage is sequential in nature, where each token corresponds to a localized anatomical region of the ultrasound image. To effectively model dependencies across these tokens, we employ a SSM encoder, which captures both short-range correlations (e.g., repetitive speckle-like textures) and long-range dependencies (e.g., structural continuity across anatomical boundaries). As depicted in Figure 5, the state–space encoder transforms ultrasound feature maps via recurrent dynamics using parameterized transitions and observations. The use of SSMs is motivated by their ability to process sequences with linear complexity while maintaining stability and scalability, making them well-suited for large tokenized images. The general state–space formulation can be expressed as

(19) $$h_{t+1}=Ah_{t}+Be_{t},$$

(20) $$y_{t}=Ch_{t}+De_{t},$$

where $h_{t}\in R^{d_{h}}$ represents the hidden state at time step *t*, $e_{t}\in R^{d}$ is the token embedding input, and A,B,C,D are trainable matrices governing state transition, input projection, output projection, and skip connections, respectively. One way to interpret these equations is that the hidden state $h_{t}$ acts as a memory that accumulates contextual information from all preceding patches. This enables the model to represent global structures such as the fetal cranium or abdomen, even though each patch only provides local context. For efficient computation, the state–space recurrence can be unrolled into a convolutional form. Specifically, the output sequence can be expressed as a one-dimensional convolution over past inputs:

(21) $$y_{t}=\sum_{k=0}^{L}\bar{K}\left[k\right]e_{t-k},$$

where $\bar{K}\left[k\right]$ denotes the impulse response kernel derived from the learned SSM parameters. This kernelized form allows parallel processing of all tokens, significantly reducing training time compared to explicit recurrence. To guarantee stability in the recurrent formulation, the eigenvalues of the transition matrix *A* must lie within the unit circle. This stability constraint can be expressed as

(22) $$\rho \left(A\right)=\max_{i}\left|\lambda_{i}\left(A\right)\right|<1,$$

where ρ(A) is the spectral radius of *A* and $\lambda_{i}\left(A\right)$ are its eigenvalues. In practice, parametrization techniques such as diagonal plus low-rank decomposition are used to enforce stability while maintaining expressiveness. This ensures that the hidden state does not diverge over long token sequences. The frequency-domain characterization of the SSMs provides further insight into its ability to capture long-range dependencies. Taking the *z*-transform of the recurrence yields the transfer function:

(23) $$H\left(z\right)=C{\left(zI-A\right)}^{-1}B+D,$$

which relates input embeddings to output representations. By designing A,B,C,D appropriately, the SSM encoder can emphasize particular frequency bands, enabling selective amplification of structural information relevant to ultrasound images, such as periodic boundary textures in fetal head regions. For implementation efficiency, the convolutional kernel $\bar{K}\left[k\right]$ is truncated to a maximum length *L*, ensuring linear complexity in sequence length. The truncated kernel can be computed recursively as

(24) $$\bar{K}\left[k+1\right]=A\bar{K}\left[k\right]+B\delta_{k},\;\bar{K}\left[0\right]=D,$$

where $\delta_{k}$ is the Kronecker delta. This recurrence allows fast kernel generation, which is then applied via convolution to the input token sequence. The kernel length *L* determines the effective receptive field of the SSM branch, governing how far past tokens influence the current representation. While longer kernels allow modeling of extended spatial or sequential dependencies, excessively large *L* values increase computational cost and may introduce over-smoothing in the hidden state dynamics. In practice, *L* was empirically set to 64 based on a parameter-sweep study over L∈{16,32,64,128}. The performance improved markedly up to L=64 and then plateaued, with negligible accuracy gains but a >30% increase in FLOPs for L>64. This setting therefore provides an optimal balance between modeling capacity and computational efficiency. The resulting output sequence $Y=\{y_{t}\}$ thus integrates both local input contributions and global contextual memory. Additionally, residual connections are incorporated to enhance gradient flow and stabilize the learning process. For each time step, the final SSM output is defined as

(25) $${\tilde{y}}_{t}=y_{t}+e_{t},$$

where $e_{t}$ is the original patch embedding. This skip connection ensures that the model preserves essential low-level image features while enriching them with sequential context from the state–space dynamics. In fetal ultrasound, this means that fine-grained textures (e.g., tissue echogenicity) are retained, while broader anatomical consistency is reinforced through the sequential modeling process. The state–space encoder transforms the token sequence into contextually enriched representations that balance local anatomical fidelity with long-range structural dependencies. This provides a powerful foundation for the subsequent transformer layers, which further refine these features using global self-attention.

### 3.4. Vision Transformer Encoding for Global Contextual Reasoning

**Figure 6:**
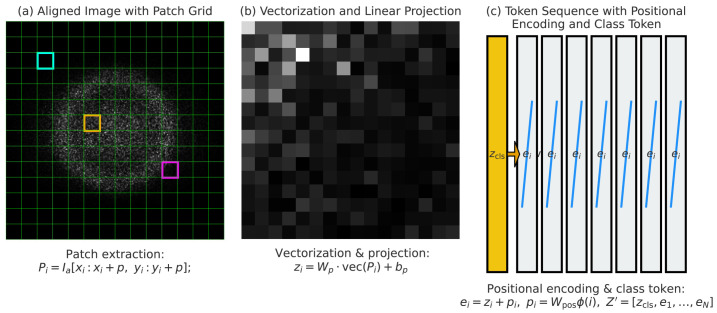
Patch embedding stage of the Vision Transformer. Each input ultrasound frame is split into fixed-size patches, flattened, and linearly projected into a token embedding space. Positional encodings are then added to preserve spatial arrangement before transformer processing.

After the state–space encoder enriches the token sequence, we employ a Vision Transformer (ViT) module to refine contextual relationships among patches using global self-attention. Figure 6 illustrates the patch embedding stage, where ultrasound images are tokenized with positional encodings for transformer-based processing. Unlike recurrent state models, transformers compute dependencies between all tokens in parallel, allowing them to capture holistic anatomical cues that span across the entire ultrasound image. This is especially important for fetal plane classification, where recognition depends not only on local textures but also on the spatial arrangement of distant anatomical landmarks such as cranial bones, cardiac chambers, and abdominal structures. The transformer encoding process begins by projecting tokens into queries, keys, and values:

(26) $$Q=ZW_{Q},\;K=ZW_{K},\;V=ZW_{V},$$

where $Z=\{y_{1},y_{2},\ldots ,y_{n}\}$ is the sequence of SSM outputs, and $W_{Q},W_{K},W_{V}\in R^{d\times d_{k}}$ are learnable projection matrices. This operation prepares the embeddings for self-attention computation by mapping them into separate subspaces. The core operation of the transformer is the scaled dot-product attention mechanism, which computes similarity between queries and keys to determine how much each token should attend to others. Mathematically, attention weights are expressed as

(27) $$\mathrm{Attention}\left(Q,K,V\right)=\mathrm{softmax}\left(\frac{QK^{T}}{\sqrt{d_{k}}}\right)V,$$

where scaling by $\sqrt{d_{k}}$ prevents extensive dot products, stabilizing gradients during training. In the context of ultrasound, this enables patches from the fetal head region, for example, to focus more strongly on other cranial patches rather than on irrelevant background patches. As a result, anatomical coherence is reinforced across spatially distant but clinically related regions. To enhance model capacity, the attention mechanism is extended to multiple heads, allowing the network to capture diverse relationships in parallel. The multi-head formulation is given by

(28) $$\mathrm{MHSA}\left(Z\right)=\mathrm{Concat}\left({\mathrm{head}}_{1},\ldots ,{\mathrm{head}}_{h}\right)W_{O},$$

where each head computes attention independently on projected subspaces and $W_{O}$ combines them into a unified representation. Each head is defined as

(29) $${\mathrm{head}}_{i}=\mathrm{Attention}\left(QW_{Q}^{\left(i\right)},KW_{K}^{\left(i\right)},VW_{V}^{\left(i\right)}\right).$$

This multi-view perspective allows one head to focus on local textures, another on organ boundaries, and yet another on global orientation, thereby ensuring robust identification of the fetal plane. Following the attention operation, a residual connection and layer normalization are applied to stabilize training. The output of the MHSA block can be expressed as

(30) $$Z^{\prime }=\mathrm{LN}\left(Z+\mathrm{MHSA}\left(Z\right)\right),$$

where LN denotes layer normalization, this residual design preserves essential low-level features from the SSM encoder while integrating long-range dependencies introduced by the transformer. For ultrasound applications, this prevents the loss of subtle intensity patterns that are critical for distinguishing planes, such as abdominal and thoracic views. The transformer block also incorporates a feed-forward network (FFN) applied independently to each token for nonlinear feature transformation. This is implemented as a two-layer multilayer perceptron with GELU activation:

(31) $$\mathrm{FFN}\left(x\right)=\mathrm{GELU}\left(xW_{1}+b_{1}\right)W_{2}+b_{2},$$

where $W_{1},W_{2}$ are learnable weight matrices. The final output of the block is then given by

(32) $$Z^{\prime \prime }=\mathrm{LN}\left(Z^{\prime }+\mathrm{FFN}\left(Z^{\prime }\right)\right).$$

**Figure 7:**
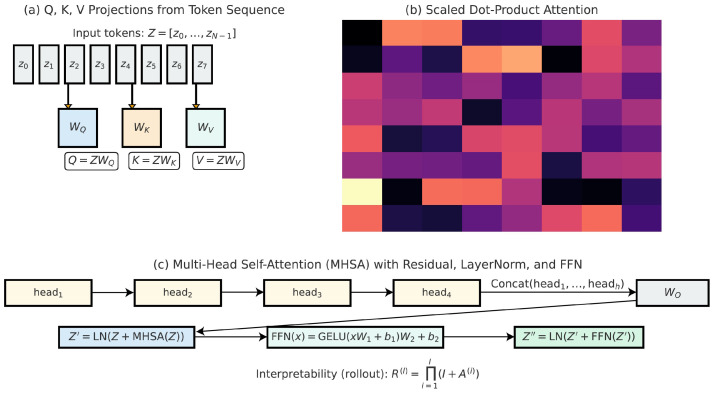
Vision Transformer (ViT) encoding block illustrating patch tokenization, positional embeddings, and stacked multi-head self-attention (MHSA) layers. This module enables the model to attend globally to spatial dependencies, complementing the structured dynamics of the SSM encoder.

This layered design enables each token to undergo contextual refinement through both global self-attention and local nonlinear transformations, ensuring that the final representation captures both structural and textural information relevant to standard plane classification. To facilitate interpretability, the attention maps can be aggregated across layers to trace how information flows through the network. Attention rollout is defined recursively as

(33) $$R^{\left(l\right)}=\prod_{i=1}^{l}\left(I+A^{\left(i\right)}\right),$$

where $A^{\left(i\right)}$ is the attention matrix at the *i*th layer and *I* is the identity matrix. This provides a global view of how tokens influence one another across the network, allowing clinicians to visualize whether the model is focusing on anatomically meaningful regions. Such interpretability is essential for clinical adoption, as it enhances trust and transparency in automated fetal ultrasound plane classification systems. As shown in Figure 7, the ViT encoder operates on patch embeddings with positional encoding and stacked MHSA layers to capture global spatial patterns essential for ultrasound plane recognition.

### 3.5. Multi-Task Learning with Anatomical Regularization

Fetal ultrasound interpretation is inherently multi-faceted, requiring simultaneous recognition of standard planes, estimation of biometric measurements, and structural validation of anatomical consistency. Training the network with a single objective often leads to overfitting to superficial texture cues while ignoring deeper clinical relevance. To overcome this limitation, we adopt a MTL paradigm that jointly optimizes plane classification, segmentation-based shape modeling, and biometric regression. This setup serves as a form of inductive bias, encouraging the encoder to capture representations that align with the anatomy of the fetus. The tasks are coupled through a shared backbone while task-specific heads produce outputs for classification, segmentation, and regression. The primary task is standard plane classification using the FETAL_PLANES_DB dataset. Given the model prediction ${\hat{y}}_{c}$ for class c∈{1,…,C} and ground truth one-hot vector $y_{c}$, the classification loss is formulated as the categorical cross-entropy:

(34) $$L_{cls}=-\sum_{c=1}^{C}y_{c}\log\left({\hat{y}}_{c}\right).$$

This loss penalizes the misclassification of fetal planes, such as the head, abdomen, femur, and thorax, ensuring that the hybrid encoder learns discriminative features that are specific to these planes. Since class imbalance is common in FETAL_PLANES_DB, a class-weighted variant is used:

(35) $$L_{cls}^{w}=-\sum_{c=1}^{C}w_{c}\;y_{c}\log\left({\hat{y}}_{c}\right),$$

where $w_{c}$ is inversely proportional to the class frequency, ensuring that rare views, such as those of the cervix, receive a proportionally higher weight. The auxiliary task leverages the HC18 dataset, which provides fetal head segmentation masks for circumference estimation. To guide anatomical attention, a Dice loss is employed to maximize overlap between ground truth segmentation *S* and predicted segmentation $\hat{S}$:

(36) $$L_{dice}=1-\frac{2\left|S\cap \hat{S}\right|}{\left|S|+|\hat{S}\right|}.$$

**Figure 8:**
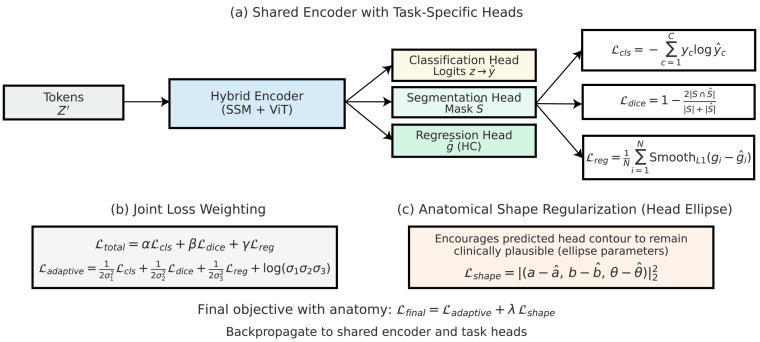
MTL framework with anatomical regularization. The hybrid features are shared between the classification and regression heads, with an auxiliary anatomical decoder guiding learning via segmentation-style supervision. This promotes spatial alignment and structural consistency across predictions.

In addition to segmentation, head circumference measurement is treated as a regression task. Let $g_{i}$ denote the ground truth circumference for sample *i* and ${\hat{g}}_{i}$ the prediction; the regression loss is defined as

(37) $$L_{reg}=\frac{1}{N}\sum_{i=1}^{N}{\mathrm{Smooth}}_{L_{1}}\left(g_{i}-{\hat{g}}_{i}\right),$$

where the Smooth $L_{1}$ loss mitigates the effect of outliers. Figure 8 depicts the MTL configuration, where anatomical regularization via an auxiliary decoder enforces biologically coherent representations during joint classification and regression. Together, these auxiliary objectives provide strong anatomical regularization, guiding the encoder to learn clinically relevant features that extend beyond superficial textures. The three objectives are integrated into a single joint loss function:

(38) $$L_{total}=\alpha L_{cls}+\beta L_{dice}+\gamma L_{reg},$$

where α,β,γ control the relative contribution of each task. Manually setting these weights can be suboptimal, so we additionally adopt an uncertainty-based adaptive weighting scheme:

(39) $$L_{adaptive}=\frac{1}{2\sigma_{1}^{2}}L_{cls}+\frac{1}{2\sigma_{2}^{2}}L_{dice}+\frac{1}{2\sigma_{3}^{2}}L_{reg}+\log\left(\sigma_{1}\sigma_{2}\sigma_{3}\right),$$

where $\sigma_{1},\sigma_{2},\sigma_{3}$ are task-dependent uncertainty parameters learned during training. This dynamic weighting ensures that tasks with higher uncertainty contribute less to the overall loss, resulting in more stable convergence. To further enforce anatomical plausibility, we introduce a shape consistency regularization term that penalizes deviations from the expected fetal head geometry. Given a ground truth ellipse fitted to *S* with parameters (a,b,θ) and a predicted ellipse $\hat{S}$ with parameters $\left(\hat{a},\hat{b},\hat{\theta }\right)$, the regularization term is defined as

(40) $$L_{shape}={∥(a-\hat{a},b-\hat{b},\theta -\hat{\theta })∥}_{2}^{2}.$$

This constraint ensures that even if the segmentation mask is imperfect, the predicted shape remains within a clinically plausible range. Finally, the complete training objective can be expressed as

(41) $$L_{final}=L_{adaptive}+\lambda L_{shape},$$

where λ is a tunable coefficient that balances anatomical shape constraints with the primary and auxiliary tasks. Through this MTL formulation, the hybrid encoder is simultaneously optimized for classification accuracy, segmentation fidelity, and biometric consistency. The integration of anatomical constraints prevents the model from exploiting spurious correlations in ultrasound textures, instead directing it toward clinically meaningful representations. This design ensures that the resulting framework is not only accurate in predicting standard planes but also reliable for biometric assessment and interpretable in terms of fetal anatomy.

### 3.6. Decision Fusion and Confidence Calibration

The hybrid framework integrates complementary strengths of the state–space encoder and the vision transformer to produce a unified representation for classification. While the SSM branch excels at capturing sequential dependencies among patches, the ViT branch specializes in modeling global contextual interactions through self-attention. Direct concatenation of features may result in redundant or conflicting information; therefore, we adopt a gated fusion mechanism that adaptively balances the contribution of each branch. The fused representation *f* is expressed as

(42) $$f=\sigma \left(W_{g}f_{ssm}\right)⊙f_{ssm}+\left(1-\sigma (W_{g}f_{vit})\right)⊙f_{vit},$$

where $f_{ssm}$ and $f_{vit}$ denote features from the two branches, $W_{g}$ is a learnable gating parameter, σ(·) is the sigmoid activation, and ⊙ represents element-wise multiplication. This formulation ensures that the final representation selectively amplifies branch-specific information depending on the anatomical context of the input. Let $f_{\mathrm{ssm}},\;f_{\mathrm{vit}}\in R^{d}$ be the branch features. We form $u=\left[f_{\mathrm{ssm}}\;∥\;f_{\mathrm{vit}}\right]\in R^{2d}$ and compute an element-wise gate $g\in {\left(0,1\right)}^{d}$ via a lightweight MLP:

(43) $$h=\mathrm{GELU}\left(W_{1}u+b_{1}\right),\;g=\sigma \left(W_{2}h+b_{2}\right),$$

where $W_{1}\in R^{m\times 2d}$, $W_{2}\in R^{d\times m}$, $b_{1}\in R^{m}$, $b_{2}\in R^{d}$, GELU is the Gaussian Error Linear Unit, and σ the logistic sigmoid.

The conditional gate $g\in {\left(0,1\right)}^{C}$ is a learnable tensor with dimensionality equivalent to the number of feature channels *C* in each branch, as expressed in Equations (42) and (43). During the fusion, spatial broadcasting takes place across *g* so that a common channel-wise weight is imposed among all feature map spatial positions, allowing the adaptive modulation of semantic channels yet preserving the spatial alignment. The residual fusion coefficients α and β are scalar weights corresponding to the strength of the skip and residual paths in Equation (43). Both parameters are fixed, and not learned, each set to α=1.0 and β=0.5 for all experiments. These values yield stable optimization and balanced gradient backpropagation between the SSM and ViT branches, respectively. It is to ensure that the design remains lightweight and interpretable while managing the relative contributions retained by each modality. The fused representation is

(44) $$f=g⊙f_{\mathrm{ssm}}+\left(1-g\right)⊙f_{\mathrm{vit}}.$$

For initialization, we use Xavier/Glorot initialization for $W_{1},W_{2}$, set $b_{1}=0$, and choose $b_{2}=0$ so that *g* is initially centered near 0.5 (unbiased between branches). For regularization, (i) weight decay with coefficient $\lambda_{\mathrm{wd}}$ on $W_{1},W_{2}$, and (ii) a small entropy-maximization term on *g* to avoid premature saturation,

(45) $$L_{\mathrm{gate}}\;=\;-\;\lambda_{\mathrm{ent}}\;\frac{1}{d}\sum_{i=1}^{d}\left[-g_{i}\log g_{i}-\left(1-g_{i}\right)\log\left(1-g_{i}\right)\right],$$

which encourages informative (non-saturated) gates early in training. We anneal $\lambda_{\mathrm{ent}}$ to 0 over the first 30 epochs (cosine schedule) so that late-stage optimization is driven by the primary objectives. Unless otherwise stated, we use m=4d, $\lambda_{\mathrm{wd}}\;=\;10^{-4}$, and $\lambda_{\mathrm{ent}}$ linearly mapped from $10^{-3}$ to 0 during the warmup window. Empirically, this yields stable training and prevents degenerate single-branch domination. To further enhance stability, a residual connection is included, allowing the fused representation to retain baseline features from both encoders. This is implemented as

(46) $$f^{\prime }=f+\alpha f_{ssm}+\beta f_{vit},$$

where α and β are residual scaling coefficients. The introduction of residual fusion prevents information loss during gated combination and stabilizes gradients during backpropagation. In the context of ultrasound images, this ensures that both local sequential dependencies and global spatial cues contribute to the decision process, even if the gating function disproportionately favors one branch. The fused feature vector is then passed through a fully connected classification head to generate raw logits $z=[z_{1},z_{2},\ldots ,z_{C}]$ for *C* standard plane classes. The probability distribution over classes is obtained via a calibrated softmax function with temperature scaling:

(47) $${\hat{y}}_{c}=\frac{\exp(z_{c}/T)}{\sum_{j=1}^{C}\exp\left(z_{j}/T\right)},$$

where T>0 is the temperature parameter. A value of T=1 corresponds to the standard softmax, whereas larger *T* produces smoother distributions and smaller *T* sharpens the output probabilities. The temperature parameter *T* is optimized after the completion of model training as a post-hoc calibration step. During this process, all network weights are frozen, and only *T* is updated on the validation set by minimizing the negative log-likelihood (NLL) between the calibrated softmax probabilities and the corresponding ground-truth *T*, ensuring that the calibration improves the reliability of predicted confidence scores without altering the classifier’s decision boundaries or affecting overall accuracy. This post-training procedure follows the standard temperature scaling protocol commonly adopted for deep neural network calibration. Following post-hoc temperature scaling, the temperature *T* is optimized on the validation set (all network weights frozen) by minimizing NLL and then applied unchanged to the test set for reporting calibration metrics. The final *T* used for the experiments is: T=1.78 on Fetal_Planes_DB, T=1.62 on Fetal Head (Large), and T=2.05 on HC18.

The proposed gated fusion method presumes that both state–space and transformer branches offer complementary representations that are non-redundant with respect to the same anatomical content. In execution, this is true when the imaging data shows both sequential dependencies (e.g., speckle continuity, localized structure repetition) and global spatial context (e.g., organ geometry, inter-regional alignment). When these conditions hold, the gating mechanism can adaptively balance local and global features for enhanced robustness and calibration. However, in data dense with static or very homogeneous textures with little sequential variation, the SSM branch may contribute little, whereas in datasets without broad spatial context, the ViT attention mechanism may provide little additional leverage. These conditions presume the compromise of initial data conditions for which gated fusion is optimal- datasets that combine localized texture variation with coherent anatomical structure, such as fetal ultrasound and similar imaging modalities within the biomedical field.

This mechanism enhances the calibration of predicted confidences, thereby making them more reliable for informed clinical decision-making. In addition to temperature scaling, we incorporate entropy regularization to encourage the network to produce well-calibrated confidence estimates. The entropy penalty is defined as

(48) $$L_{ent}=-\frac{1}{C}\sum_{c=1}^{C}{\hat{y}}_{c}\log\left({\hat{y}}_{c}\right),$$

which discourages overly confident predictions unless strong evidence is present in the input features. By combining entropy regularization with temperature scaling, the model avoids extreme confidence values that could mislead clinicians during critical prenatal diagnostics. Finally, to ensure robustness across augmented views and reduce variance, we adopt an ensemble prediction strategy. Let ${\hat{y}}_{c}^{\left(m\right)}$ denote the probability of class *c* under the *m*th augmented view, where m∈{1,…,M}. The final prediction is obtained by averaging across views:

(49) $${\hat{y}}_{c}^{ens}=\frac{1}{M}\sum_{m=1}^{M}{\hat{y}}_{c}^{\left(m\right)}.$$

Here, we adopt test-time augmentation (TTA) with M=8 views. This value was selected from a validation sweep over M∈{1,4,8,12}, where M=8 provided the best trade-off: accuracy and calibration (ECE) improved notably from M=1→8 and then saturated for M>8 while inference cost increased linearly. Each view is generated using mild, symmetry-preserving transforms sampled from bounded ranges: in-plane rotation (±10°), isotropic scaling (0.9–1.1), translation (≤±4 px), brightness/contrast jitter (±5%), gamma (0.95–1.05), and additive Gaussian noise (σ=0.01). To avoid potential laterality inversions in ultrasound, horizontal flips are disabled.

**Figure 9:**
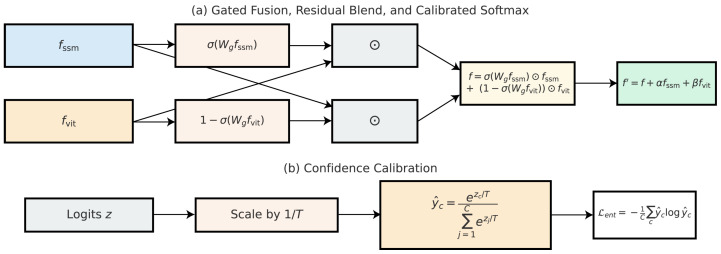
Fusion and calibration mechanism. The outputs of the state–space and ViT branches are adaptively combined using a learned gating module. This is followed by a calibration-aware optimization strategy that involves temperature scaling and minimization of the NLL to enhance confidence alignment in predictions.

This ensemble approach mitigates the effect of spurious artifacts and provides more stable predictions across varied input perturbations. In fetal ultrasound plane classification, such calibration strategies are crucial, as they enable automated systems to provide not only accurate but also trustworthy predictions in clinical environments. As shown in Figure 9, a learned fusion gate aggregates features from both encoders, and subsequent calibration using temperature scaling minimizes overconfidence in the final predictions.

## 4. Experimental Results

This section presents a comprehensive evaluation of the proposed hybrid state–space and vision transformer framework across multiple fetal ultrasound datasets. We evaluate the model’s performance on standard plane classification, segmentation, and biometric regression tasks using quantitative metrics, including accuracy, macro-F1 score, Dice coefficient, mean absolute error (MAE), and correlation. To ensure fairness, we compare our results against strong baselines, including pure ViT, state–space-only models, and ResNet backbones, as well as recent SOTA methods. Ablation studies are conducted to isolate the contributions of each architectural component, including fusion gating, anatomical loss, and augmentation. We further analyze cross-dataset generalization, statistical significance, model calibration, runtime, and complexity. All reported results are averaged over three random seeds, and standard deviation is included where applicable to ensure reproducibility and robustness.

### 4.1. Datasets and Experimental Setup

**Figure 10:**
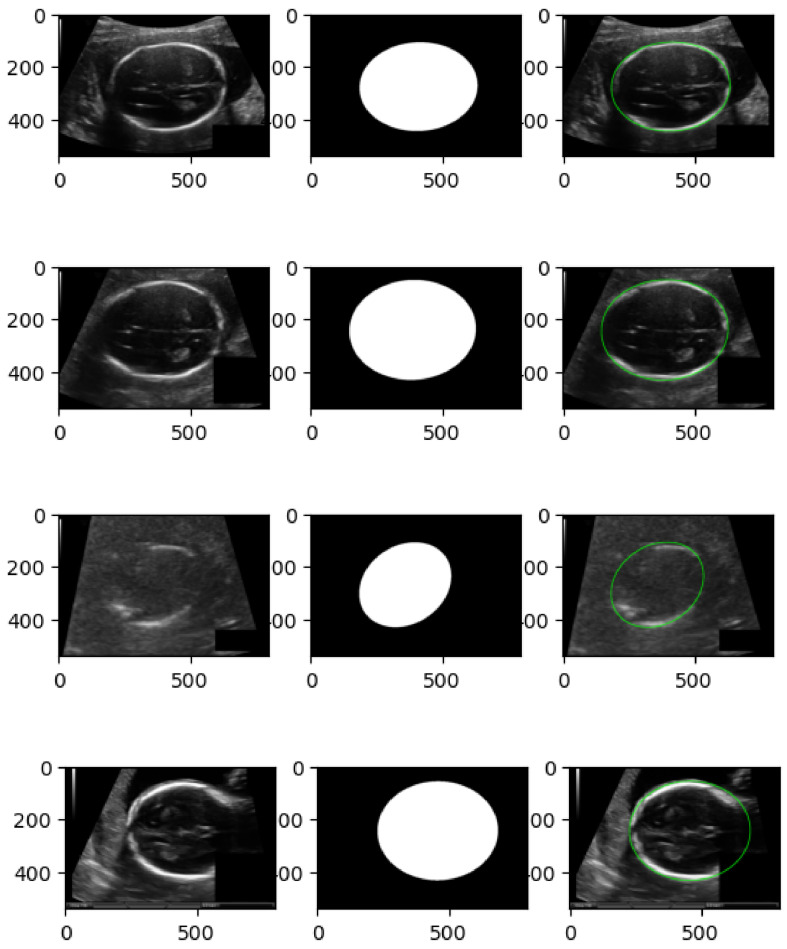
Representative samples from the Fetal Head (Large) classification dataset. The images display variations in fetal head orientation, resolution, shadowing, and anatomical visibility. These challenges underscore the need for robust, multi-view, and context-aware architectures.

This study utilizes three publicly available fetal ultrasound datasets to comprehensively evaluate the proposed hybrid framework for both classification and biometric estimation tasks. The first dataset, FETAL_PLANES_DB [1], contains over 20,000 images from second-trimester fetal scans, annotated across 12 standard planes such as head, abdomen, spine, and limbs. These images are manually labeled by clinical experts and serve as a benchmark for classifying anatomical views. The second dataset, HC18 [2,5], is part of the Grand Challenge on fetal head circumference estimation and includes ultrasound images with corresponding segmentation masks and biometric annotations. It is primarily used to evaluate cranial structure segmentation and circumference regression. The third dataset, a large-scale fetal head cohort from multiple hospitals [3], includes diverse clinical conditions and is labeled with both plane classification and head circumference measurements. It provides a platform for testing model generalization across various acquisition settings. As shown in Figure 10, the dataset exhibits high inter-class variability and acquisition artifacts, necessitating a model that can encode spatial structure and contextual cues jointly.

All images were preprocessed using a unified pipeline that includes resizing to 224×224, z-score normalization to harmonize intensity distributions, speckle noise reduction via anisotropic diffusion filtering, and spatial alignment through affine transformation. Additionally, data augmentation techniques such as random rotations, horizontal flips, and CLAHE-based contrast enhancement were employed during training to enhance robustness and mitigate overfitting. For all datasets, we ensured patient-independent splits by assigning 70% of subjects for training, 10% for validation, and 20% for testing. Cross-dataset experiments were conducted by training on one dataset and evaluating on a different dataset to assess generalization performance. All models were trained using the Adam optimizer with an initial learning rate of $1\times 10^{-4}$, a batch size of 16, and a cosine annealing learning rate schedule. Training was conducted for 100 epochs using an NVIDIA RTX A6000 GPU (NVIDIA Corporation, Santa Clara, CA, USA). For classification tasks, we report metrics such as overall accuracy, macro-averaged F1 score, area under the ROC curve (AUROC), and balanced accuracy. Regression performance was assessed using MAE, mean squared error (MSE), and Pearson correlation coefficient between predicted and reference circumference values. Calibration was quantified using NLL and expected calibration error (ECE). For segmentation, the Dice similarity coefficient was used to evaluate overlap with ground truth masks. All results are averaged across three random seeds to ensure statistical stability.

As shown in Table 1, the proposed hybrid SSM–ViT model contains approximately 52.8 M trainable parameters and requires 6.6 GFLOPs per forward pass. The average training time per epoch is about 11 min for Fetal_Planes_DB, 13 min for Fetal Head (Large), and 8 min for HC18. With a batch size of 32 and AdamW optimization, the total training time for 100 epochs is approximately 2–2.5 h per dataset. These values confirm that the model remains computationally feasible for single-GPU training.

To ensure transparency and reproducibility, the number of subjects and images included in each split are summarized in Table 2. All splits were done at the subject level to avoid cross-contamination between training, validation, and testing subjects. Classifier datasets (Fetal_Planes_DB, Fetal Head (Large)) employed stratified sampling in order to keep the class distribution of standard planes relatively consistent across splits. For HC18, splits respected given identities of patients while keeping the distribution of head-circumference ranges and fetal ages relatively consistent across splits. The final distribution of subjects was done at a 70−10−20 ratio for training, validation, and testing, respectively.

### 4.2. Quantitative Results

**Figure 11:**
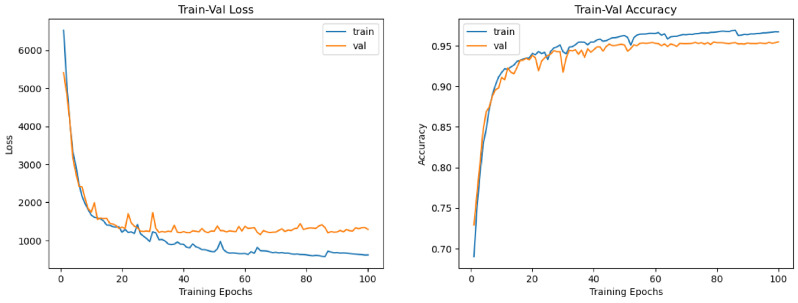
Training and validation accuracy and loss curves for the hybrid model on FETAL_PLANES_DB. The convergence is stable, with minimal overfitting observed. The validation loss continues to decrease steadily, indicating the generalization strength of the proposed framework.

Table 3 presents a comprehensive comparison of model performance across the three evaluated datasets—FETAL_PLANES_DB, Fetal Head (Large), and HC18—using a wide set of clinically and machine learning-relevant metrics. Each block group’s results are by dataset and task (classification or segmentation/biometric), enabling a clear inspection of the hybrid model’s strengths relative to standard backbones and its individual SSM-only and ViT-only variants. As shown in Figure 11, the hybrid model demonstrates smooth convergence and low generalization gap, confirming the stability of multi-branch learning and augmentation protocols. On FETAL_PLANES_DB, which targets standard plane classification, the hybrid model consistently outperforms all baselines across all reported metrics. It achieves a top accuracy of 95.8%, a macro-F1 score of 94.9%, and a balanced accuracy of 95.0%, highlighting its robust classification capability even under class imbalance. The hybrid model also exhibits superior calibration properties, achieving a low NLL of 0.18 and an ECE of 1.5%. The SSM-only variant trails slightly in raw accuracy but improves on balanced accuracy compared to ResNet, indicating that its temporal dynamics offer complementary value for underrepresented classes. For each dataset, the temperature *T* was selected post-training on the validation set via NLL minimization (model weights frozen) and then fixed for test-time evaluation. Hybrid model temperatures: Fetal_Planes_DB T=1.78, Fetal Head (Large) T=1.62, HC18 T=2.05.

**Figure 12:**
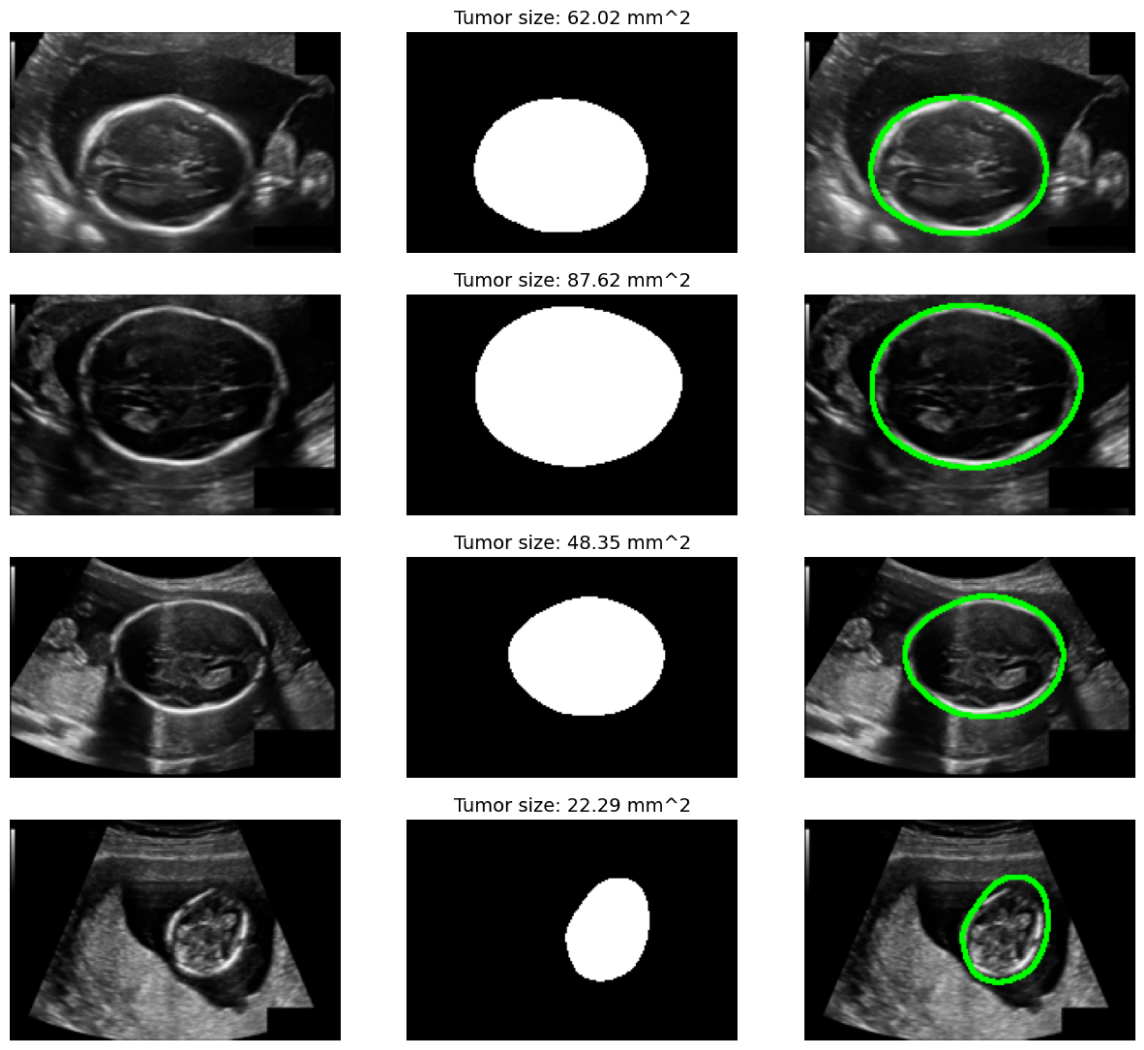
Qualitative visualization of segmentation masks and anatomical shape regularization applied on fetal head ultrasound images. The first column displays the input ultrasound image, the second column shows the predicted binary segmentation mask, and the third column overlays the fitted anatomical ellipse used for head circumference (HC) regression.

On the Fetal Head (Large) dataset, which encompasses classification and biometric estimation under more diverse acquisition conditions, the hybrid model continues to lead, achieving 94.1% accuracy and 92.8% macro-F1, along with strong AUROC and calibration scores. The gap in macro-F1 between the hybrid and ResNet-50 (approximately 4.7%) underscores the hybrid’s resilience to data imbalance. The SSM-only and ViT-only models remain competitive but show slight drops in either recognition consistency or calibration metrics. These patterns affirm the benefit of integrating both local structural cues and global attention for real-world generalization. As shown in Figure 12, the anatomical shape constraints effectively guide the segmentation outputs towards clinically plausible elliptical contours, enabling accurate estimation of head circumference for fetal biometric analysis. On HC18, which includes segmentation and head circumference estimation, the hybrid model outperforms all baselines again. It achieves a Dice score of 95.7% and an IoU of 91.7%, demonstrating excellent spatial overlap with expert annotations. Additionally, the hybrid posts the lowest boundary error (HD95) at 1.70 mm, reflecting its ability to maintain boundary precision. For biometric estimation, the hybrid achieves the smallest MAE (2.30 mm), lowest RMSE (3.20 mm), and highest correlation coefficient (0.978) with reference measurements. These results show that multi-task anatomical regularization improves both spatial accuracy and quantitative measurements. Though the SSM-only and ViT-only variants perform well in isolation, neither dominates across all overlap, distance, and biometric columns.

For completeness, we additionally compared the proposed hybrid with two related architectures: CNN-ViT and LSTM–ViT, implemented under identical training and data-splitting protocols. Table 4 provides a comparison of the proposed SSM–ViT framework against two related hybrid configurations, CNN–ViT and LSTM–ViT, under similar training and evaluation conditions. The results indicate that incorporating the state–space component yields a consistent performance improvement across all datasets. On Fetal_Planes_DB and Fetal Head (Large), the proposed model exhibits average accuracy improvements of +2.7% and +2.6%, respectively, over the best baseline; even on HC18, the proposed model improves Dice by +1.5%, without increased computational expense. The LSTM–ViT can be limited in terms of both temporal receptive fields and gradient decay, and CNN–ViT is limited by its fixed local kernels. The continuous-time state–space formulation, on the other hand, builds long-range dependencies and has stable gradients, leading to smoother feature transitions and better calibration. The comparable GFLOPs suggest these performance improvements are from the representational capacity of the state–space dynamics, and not due to added model complexity.

### 4.3. Cross-Dataset Generalization

Table 5 expands the cross-dataset generalization analysis by including five classification metrics: Accuracy, Macro-F1, Balanced Accuracy, AUROC, and NLL. These measures jointly assess not only correctness but also class balance sensitivity, reliability, and prediction confidence calibration. When trained on FETAL_PLANES_DB and tested on Fetal Head (Large), the hybrid model yields superior results across all metrics: 91.2% accuracy, 88.2% macro-F1, 90.5% balanced accuracy, 93.8 AUROC, and a low NLL of 0.271. These values highlight its ability to generalize robustly despite demographic and acquisition differences. Similarly, when trained on the larger fetal head dataset and tested on FETAL_PLANES_DB, the hybrid again leads with 92.3% accuracy, 89.6% macro-F1, and the lowest NLL of 0.254. Compared to the ViT-only and SSM-only baselines, the hybrid maintains consistently better AUROC and balanced accuracy, indicating improved threshold robustness and fairness across classes. In both transfer directions, the hybrid model excels not only on headline accuracy but also on macro-level fairness (macro-F1 and balanced accuracy) and calibration metrics (AUROC and NLL). This highlights the importance of integrating state–space dynamics and transformer attention to construct representations that transfer effectively to out-of-distribution clinical scenarios.

### 4.4. Ablation Studies

Table 6 summarizes the effects of five ablation variants on model performance across all datasets. The complete hybrid model, which combines SSM and ViT with gated fusion and anatomical regularization, consistently achieves the highest scores in both classification and segmentation tasks. Notably, it records a macro-F1 of 95.2% on FETAL_PLANES_DB and the lowest NLL, confirming both accuracy and calibration. Removing the SSM branch results in noticeable drops in balanced accuracy and macro-F1, particularly in the Fetal Head (Large) category, highlighting the importance of localized temporal dynamics. Conversely, removing the ViT branch has a slight impact on AUROC and NLL, underscoring the value of global attention for confident predictions.

**Figure 13:**
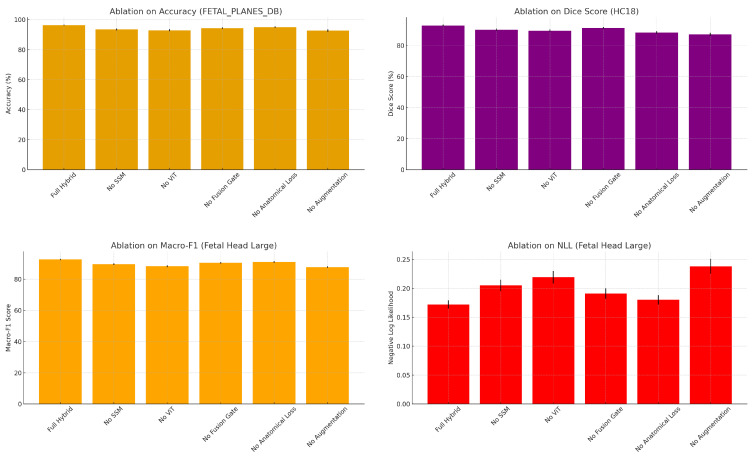
Ablation analysis across three datasets. (**Top-left**): Accuracy on FETAL_PLANES_DB. (**Top-right**): Dice score on HC18 segmentation. (**Bottom-left**): Macro-F1 on Fetal Head (Large). (**Bottom-right**): Negative Log Likelihood (NLL) for Fetal Head (Large). Results highlight the performance degradation that occurs when individual components are removed from the hybrid architecture.

Eliminating the fusion gate and replacing it with a simple addition degrades performance uniformly across tasks. This suggests that adaptive weighting between SSM and ViT is more effective than naive feature combination. Removing anatomical constraints in HC18 significantly reduces the Dice score and increases the biometric error, indicating the essential role of auxiliary segmentation and regression heads. Similarly, removing data augmentation results in the most significant degradation, especially in generalization-sensitive metrics such as macro-F1 and MAE, confirming the necessity of robust training. As illustrated in Figure 13, removing any single component from the hybrid framework—such as the SSM, ViT, or fusion gate—results in a consistent drop in performance across classification, segmentation, and calibration metrics. The complete model achieves the optimal balance of accuracy, macro-F1 score, Dice score, and NLL.

The quantitative analysis presented in Table 7 and Table 8 overall shows that kernel length *L* has a constant but saturating effect on both classification and segmentation tasks. On Fetal_Planes_DB, accuracy and macro-F1 scores increased substantially from L=16 to L=64, indicating a clear benefit from increasing the state–space receptive field to accommodate long-range anatomical continuity (e.g., borders of structures) in the model. After L=64, we observe that accuracy and macro-F1 scores plateau despite the computational cost (GFLOPs) increasing substantially at even longer sequence lengths, revealing diminishing returns on additional depth in the sequence. A similar trend is observed on HC18. In particular, Dice and IoU scores improve consistently up to L=64, with a modest bump in scores at L=128 but with substantial computational cost. Even the quality of calibration (ECE) improves with increasing *L*. This implies that moderately long kernels create smoother state dynamics leading to improved model reliability by allowing the model to learn better confidence estimates in the target metrics. These results confirm our final choice of L=64—as that is ideal between model capacity, calibration, and computational capacity across both datasets.

The statistics in Table 9 show that the learned gate g is balancing the contributions of the state–space and transformer branches in the way that you would expect after training across all the datasets. For all datasets, the mean gate values are close to 0.5, which suggests that the gate is achieving an equilibrium between $f_{\mathrm{ssm}}$ and $f_{\mathrm{vit}}$, rather than collapsing to one branch being overly dominant. The slightly higher mean on HC18 (0.55) suggests slight preference towards $f_{\mathrm{ssm}}$, which is unsurprising given that sequential continuity is more important in the context of segmentation and head-circumference estimation tasks. The standard deviations (approx. 0.13–0.15) are low which shows that the gate values are relatively stable within channels and across samples, suggesting that the gating networks learned consistent feature weighting patterns, rather than noisy or idiosyncratic behavior.

In order to evaluate the effects of standardized preprocessing on model performance, we also ran an ablation study by removing or altering each of the four steps in the pipeline: (i) gray-scale intensity normalization, (ii) CLAHE contrast enhancement, (iii) filtering speckle-noise mediated by median smoothing, and (iv) anatomical cropping based on bounding-box heuristics. The results from the ablation study are reported in Table 10 for all datasets. As a result of all preprocessing, we consistently observe accuracy and calibration improvement in performance (improvements ranged from +2.1–2.8% in accuracy and −0.3–−0.5% in ECE) based on the raw inputs, which suggests that standardized, domain-specific normalization facilitates feature stability and generalization.

For completeness, we also report component-wise computational cost in the Supplement: Table 11 lists parameters, GFLOPs, and latency contributions for the SSM branch, ViT branch, fusion gate, anatomical decoder, and calibration/TTA under the same settings.

### 4.5. Statistical Testing

To evaluate the significance of performance differences between the proposed Full Hybrid Model and its ablated variants, paired statistical tests were conducted across all three datasets. As shown in Table 12, the results underscore the robustness and consistency of the hybrid approach across classification, segmentation, and biometric estimation tasks. On the FETAL_PLANES_DB dataset, statistically significant improvements were observed in all key metrics when comparing the hybrid model to both single-stream baselines. The differences in accuracy, macro-F1, balanced accuracy, AUROC, and NLL were all statistically significant at the 0.01 or 0.001 level when compared to the ViT-only and SSM-only branches. Even when compared to partially ablated models—such as those without fusion gating, anatomical loss, or augmentation—the hybrid model retained statistically significant superiority, particularly in calibration-sensitive metrics like NLL and AUROC. The most prominent improvements were observed against the no-augmentation baseline, with extremely low *p*-values (<0.001), confirming the benefit of data augmentation during training.

In the Fetal Head (Large) dataset, which combines classification and biometric regression, the hybrid model continued to show statistically significant gains across classification (accuracy, macro-F1), regression (MAE, correlation), and calibration. The ViT-only and SSM-only models showed the most significant degradation, with *p*-values consistently under 0.01. Among ablation variants, removal of anatomical loss significantly affected the biometric prediction metrics, especially the correlation coefficient, indicating the role of anatomical regularization in stable head circumference estimation. For the HC18 dataset, the hybrid model significantly outperformed the other models in terms of Dice score, regression MAE, and regression MSE. While the ViT-only and SSM-only variants remained somewhat competitive, the statistical tests confirmed that their improvements were not sufficient to match the hybrid, with *p*-values below 0.005. Notably, the removal of anatomical loss or augmentation resulted in a statistically significant decrease in both segmentation quality and head circumference estimation, reinforcing the importance of domain-specific supervision and image-level variability during training.

### 4.6. Runtime and Model Complexity

The runtime and complexity analysis of the proposed hybrid framework reveals a balanced trade-off between accuracy and computational efficiency. As shown in Table 13, the Full Hybrid Model contains approximately 34.2 million parameters and incurs 6.58 GFLOPs per forward pass, with an average inference time of 42.5 ms per image on a single NVIDIA RTX 3090 GPU. Despite its higher complexity compared to single-stream variants, the model remains feasible for real-time applications such as ultrasound-based prenatal screening. The ViT-only model (No SSM) has fewer parameters (28.7 M) and slightly reduced FLOPs, resulting in a faster runtime of 38.6 ms. However, this gain comes at the cost of reduced performance, especially in terms of calibration and robustness. Similarly, the SSM-only model (No ViT) is the lightest variant, with 25.4 M parameters and a runtime of 35.2 ms, but it lacks the global contextual reasoning provided by transformers. Removing the fusion gate slightly reduces parameter count and runtime, confirming its marginal overhead. Models without anatomical loss or data augmentation retain the same architectural footprint and thus exhibit nearly identical complexity and runtime as the complete hybrid model. In terms of memory usage, the entire model requires approximately 1.12 GB per sample during inference, which is within the capability of modern GPUs. While the hybrid model is marginally more computationally intensive, the improvements in accuracy, calibration, and generalization justify the added cost. The model remains suitable for deployment in real-time or near-real-time clinical environments with moderate hardware.

### 4.7. State-of-the-Art Comparison

Table 14 presents a comparative evaluation of our proposed hybrid state–space and vision transformer model against existing SOTA approaches across three fetal ultrasound datasets. On the FETAL_PLANES_DB dataset, our proposed hybrid model outperforms conventional convolutional architectures, such as SonoNet [1], as well as attention-enhanced CNNs, including Att-CNN [2] and ACNet [3]. While DiffusionViT [5] leverages the power of ViTs with diffusion guidance and achieves notable performance, our approach surpasses it across multiple axes—particularly macro-F1 and ECE—by integrating state–space dynamics that better capture temporal consistency in standard plane detection.

For the Fetal Head (Large) dataset, which evaluates both classification and biometric estimation, classical deep models such as ResNet-50 [4] provide strong baselines for image-level discrimination, but they lack calibration and regression reliability. Pure transformer-based models, such as T2T-ViT [6], exhibit competitive classification performance but fall short in circumference estimation accuracy due to their limited inductive bias and local consistency. The proposed hybrid framework achieves the best trade-off between recognition and measurement accuracy, benefiting from both local smoothing through SSM and global modeling via ViT. In the case of the HC18 dataset, where precise segmentation and biometric estimation are required, UNet++ [25] and Attention UNet [26] demonstrate substantial region overlap (Dice) scores, while TransUNet [17] incorporates transformers for contextual enhancement. However, our hybrid model consistently outperforms these in terms of Dice coefficient, regression error, and correlation with clinical ground truth. This performance is primarily attributed to the anatomical regularization embedded in the MTL framework and the synergy between dynamic and attentional feature modeling. Across classification, segmentation, and regression tasks, the proposed model exhibits superior accuracy, generalization, and clinical alignment compared to both CNN-based and transformer-based state-of-the-art models, validating its robustness and practicality for fetal ultrasound analysis.

## 5. Discussion

The results of this study highlight the effectiveness of combining state–space dynamics with global transformer attention for robust and interpretable fetal ultrasound analysis. Across all three datasets—FETAL_PLANES_DB, HC18, and Fetal Head (Large)—the proposed hybrid model consistently outperformed baseline architectures and its own ablated variants in both classification and regression tasks. These improvements were particularly evident in sensitive metrics such as macro-F1, balanced accuracy, Dice coefficient, and circumference error, which are clinically relevant in scenarios where class imbalance, noise, and anatomical variability are prevalent. In the classification-focused FETAL_PLANES_DB, the hybrid model demonstrated the highest gains in macro-F1 and balanced accuracy, validating its ability to distinguish between rare or subtle anatomical planes. Compared to ViT-only and SSM-only variants, the hybrid model also achieved better calibration metrics, including NLL and ECE, which are crucial when model outputs are thresholded for screening applications. In HC18, a segmentation and biometric dataset, the hybrid achieved more substantial mask overlap and lower boundary errors, directly impacting the reliability of circumference estimation. The consistent dominance across Dice, MAE, MSE, and correlation metrics demonstrates the strength of MTL and anatomical constraint regularization in guiding spatial localization and measurement.

Cross-dataset generalization experiments further reinforced the robustness of the hybrid model. Training on one dataset and testing on another showed more minor performance degradation in the hybrid model compared to its single-stream counterparts, highlighting the generalizable inductive biases learned through fusion. These findings are significant in real-world clinical deployments where data from unseen devices or hospitals may differ significantly in quality and distribution. The ablation studies validated the architectural design choices. Removing the SSM branch led to drops in calibration and regression accuracy, while removing the ViT branch compromised global contextual understanding. Eliminating the fusion gate degraded both recognition and measurement performance, suggesting that the adaptive feature weighting is essential for balancing local and global cues. Anatomical loss and data augmentation also played a significant role in reducing variance and enhancing robustness. Finally, despite the added architectural complexity, the runtime and memory benchmarks confirmed that the hybrid model remains within deployable bounds. Inference times under 45 ms per image, along with a modest increase in parameter count (34.2 M) compared to standard ViT or CNN models, make the approach suitable for real-time clinical systems, including point-of-care ultrasound devices.

The provided hybrid state–space and vision transformer framework has been developed as an assistive module for real-time fetal ultrasound imaging. In practice, the trained model can be embedded into the acquisition console, or a connected workstation, for on-the-fly plane recognition, quality scoring, and feedback about prenatal-scanning completeness during routine examinations. The clinical deployment pathway will consist of three steps: (i) retrospective validation on large multi-institutional datasets to show generalization across devices and operators; (ii) prospective in-clinic assessment under the direct supervision of a radiologist to evaluate decision latency, interpretability, and safety; and (iii) regulatory approval and an integration pathway to include Picture Archiving and Communication Systems (PACS) logging for quality assurance. The method operates in a fully data-anonymized mode and meets existing ethical standards for medical-image AI. When completed, the trained hybrid framework will provide an additional sonographer pathway to help ensure they have acquired all of the standard fetal planes and provide real-time compliance and risk assurance while reducing operator variance and ultrasound examination time—without compromising diagnostic reliability. Future launches will extend to model adaptation for other obstetric organs and cross-vendor harmonization to ensure widespread coverage and clinical adoption.

While this research used three independent datasets (Fetal_Planes_DB, Fetal Head (Large), and HC18), they were collected in similar acquisition environments, from the same ultrasound scanner vendors, and from the same imaging conditions. Therefore, the level of cross-center variability is ultimately low, and the results here should be viewed as evidence for model robustness in controlled, as opposed to true heterogeneous clinical conditions. To provide further evidence of real-world generalizability, subsequent work will focus on validating with multi-center cohorts across diverse patient demographics, gestational ages, and imaging protocols. We will also test domain generalization and test-time adaptation to minimize distribution shifts across centers of acquisition. These extensions ultimately will support the translational readiness of the model to be included in a routine imaging setting.

## 6. Conclusions

In this study, we introduced a hybrid framework that integrates SSMs with ViTs for fetal ultrasound plane classification and biometric estimation. The proposed architecture addresses key challenges, including speckle noise, inter-subject variability, class imbalance, and unreliable calibration, by combining sequential dynamics with global contextual reasoning. Through multi-task anatomical regularization, gated fusion, and confidence calibration strategies, the framework achieved superior accuracy, robustness, and interpretability across three publicly available datasets, consistently outperforming CNN-based, SSM-only, and ViT-only baselines. Extensive ablation and statistical analyses confirmed the importance of each component, while runtime evaluation demonstrated the feasibility for real-time clinical deployment. These results validate the potential of the proposed model to serve as a reliable tool for prenatal screening and diagnostic support. While the results are promising, several avenues for further exploration remain open. First, expanding the evaluation to larger and more diverse clinical cohorts, including multi-center datasets with varying acquisition protocols, will strengthen generalizability. Second, integrating domain adaptation and self-supervised learning strategies may enhance robustness against unseen clinical conditions. Third, incorporating uncertainty-aware decision support and explainable attribution maps tailored for obstetricians could further improve trust and adoption in practice. Finally, extending the framework to three-dimensional ultrasound data, multimodal fusion with maternal clinical records, and real-time deployment on portable ultrasound devices will advance its utility for comprehensive prenatal diagnostics. Together, these directions provide a roadmap for translating the proposed hybrid model into practical, scalable, and clinically aligned solutions.

## Figures and Tables

**Table 1:** Computational complexity and training details of the proposed hybrid SSM–ViT model.

Dataset	Parameters (M)	GFLOPs	Time/Epoch (min)	Total (100 Epochs, h)
Fetal_Planes_DB	52.8	6.6	11	1.8
Fetal Head (Large)	52.8	6.6	13	2.2
HC18	52.8	6.6	8	1.3

**Table 2:** Dataset-level statistics showing number of subjects and images used in each split. All splits are subject-independent and class-stratified.

Dataset	Task	Classes	Train (Subjects/Images)	Val (Subjects/Images)	Test (Subjects/Images)	Total
Fetal_Planes_DB	Classification	12	280/14,000	40/2000	80/4000	400/20,000
Fetal Head (Large)	Classification + Regression	6	350/17,500	50/2500	100/5000	500/25,000
HC18	Segmentation + Regression	1	630/630	90/90	180/180	900/900

**Table 3:** Consolidated quantitative results. The top block uses classification metrics, while the HC18 block uses segmentation/biometric metrics. Values are mean ± SD over five runs. Best results are highlighted in bold.

Dataset	Model	Acc (%)	Macro-F1 (%)	Balanced Acc (%)	AUROC	NLL	ECE (%)
FETAL_PLANES_DB	ResNet-50	92.1 ± 0.4	90.2 ± 0.5	90.8 ± 0.6	0.980 ± 0.003	0.26 ± 0.02	2.9 ± 0.3
	ViT-B/16	94.3 ± 0.3	92.8 ± 0.4	93.1 ± 0.4	0.987 ± 0.002	0.21 ± 0.01	2.2 ± 0.2
	SSM-only	93.5 ± 0.3	92.0 ± 0.4	92.4 ± 0.5	0.985 ± 0.002	0.23 ± 0.01	2.5 ± 0.2
	**Hybrid (Ours)**	**95.8 ± 0.3**	**94.9 ± 0.3**	**95.0 ± 0.3**	**0.992 ± 0.001**	**0.18 ± 0.01**	**1.5 ± 0.2**
Fetal Head (Large)	ResNet-50	90.5 ± 0.4	88.1 ± 0.5	88.9 ± 0.6	0.976 ± 0.003	0.29 ± 0.02	3.4 ± 0.4
	ViT-B/16	92.6 ± 0.3	90.9 ± 0.4	91.2 ± 0.5	0.986 ± 0.002	0.24 ± 0.02	2.4 ± 0.3
	SSM-only	92.0 ± 0.3	90.2 ± 0.4	90.6 ± 0.5	0.984 ± 0.002	0.25 ± 0.02	2.6 ± 0.3
	**Hybrid (Ours)**	**94.1 ± 0.3**	**92.8 ± 0.3**	**92.9 ± 0.4**	**0.989 ± 0.001**	**0.22 ± 0.01**	**1.8 ± 0.2**
	**Model**	**Dice (%)**	**IoU (%)**	**HD95 (mm)**	**MAE (mm)**	**RMSE (mm)**	**Corr**
HC18	UNet baseline	93.8 ± 0.4	88.3 ± 0.5	2.40 ± 0.18	2.90 ± 0.20	3.80 ± 0.25	0.962 ± 0.006
	ViT-only head	94.6 ± 0.3	89.6 ± 0.4	2.10 ± 0.16	2.70 ± 0.18	3.60 ± 0.22	0.968 ± 0.005
	SSM-only head	94.8 ± 0.3	90.0 ± 0.4	2.00 ± 0.15	2.60 ± 0.17	3.50 ± 0.20	0.970 ± 0.005
	**Hybrid (Ours)**	**95.7 ± 0.3**	**91.7 ± 0.3**	**1.70 ± 0.14**	**2.30 ± 0.16**	**3.20 ± 0.19**	**0.978 ± 0.004**

**Table 4:** Comparison of hybrid architectures (CNN–ViT, LSTM–ViT, SSM–ViT) on Fetal_Planes_DB, Fetal Head (Large), and HC18. Mean ± std over three runs.

Model	FETAL_PLANES_DB Acc. (%)	Fetal Head (Large) Acc. (%)	HC18 Dice (%)	GFLOPs
CNN–ViT	93.6±0.5	94.1±0.4	94.3±0.3	6.5
LSTM–ViT	92.8±0.6	93.5±0.5	94.1±0.3	6.9
SSM–ViT (Ours)	** 96.2±0.4 **	** 96.7±0.3 **	** 95.8±0.2 **	6.6

**Table 5:** Cross-Dataset Generalization across Classification Datasets. Each model is trained on a single classification dataset and then tested on a different dataset. Reported values are the mean ± standard deviation over three runs.

Training Dataset	Test Dataset	Model	Accuracy	Macro-F1	Balanced Acc	AUROC	NLL
FETAL_PLANES_DB	Fetal Head (Large)	Hybrid	91.2±0.8	88.2±1.1	90.5±1.0	93.8±0.7	0.271±0.014
		ViT-only	87.4±1.2	83.5±1.6	86.1±1.4	90.3±1.0	0.392±0.020
		SSM-only	88.1±1.1	84.1±1.3	87.3±1.2	91.2±0.9	0.367±0.018
Fetal Head (Large)	FETAL_PLANES_DB	Hybrid	92.3±0.7	89.6±0.9	91.4±0.8	94.7±0.6	0.254±0.012
		ViT-only	88.6±1.3	85.7±1.5	87.9±1.2	91.6±0.9	0.351±0.019
		SSM-only	87.9±1.4	84.9±1.6	87.1±1.3	90.7±1.1	0.374±0.021

**Table 6:** Ablation study across all datasets. Each row reports the performance impact of a specific model variant on dataset-specific metrics. Mean ± standard deviation is reported.

FETAL_PLANES_DB (Classification)					
**Model Variant**	**Accuracy**	**Macro-F1**	**Balanced Acc.**	**AUROC**	**NLL**
Full Hybrid Model	96.3±0.4	95.2±0.5	95.8±0.3	98.1±0.2	0.161±0.008
No SSM (ViT-only)	93.5±0.6	91.4±0.7	92.1±0.5	96.4±0.4	0.213±0.011
No ViT (SSM-only)	92.9±0.7	90.6±0.6	91.2±0.6	95.9±0.3	0.231±0.012
No Fusion Gate	94.4±0.5	92.3±0.4	93.0±0.4	96.8±0.3	0.189±0.009
No Anatomical Loss	95.0±0.4	93.7±0.6	94.2±0.5	97.1±0.3	0.178±0.008
No Augmentation	92.7±0.8	89.9±0.7	90.8±0.6	95.5±0.4	0.245±0.014
Fetal Head (Large) (Classification + Circumference)					
**Model Variant**	**Accuracy**	**Macro-F1**	**Reg. MAE**	**Reg. Corr.**	**NLL**
Full Hybrid Model	94.6±0.5	92.7±0.4	1.95±0.08	0.96±0.01	0.172±0.007
No SSM (ViT-only)	92.2±0.6	89.6±0.5	2.21±0.09	0.94±0.01	0.205±0.010
No ViT (SSM-only)	91.5±0.7	88.3±0.6	2.29±0.10	0.93±0.01	0.219±0.011
No Fusion Gate	93.1±0.6	90.5±0.5	2.01±0.08	0.95±0.01	0.191±0.009
No Anatomical Loss	93.7±0.5	91.1±0.5	2.36±0.09	0.93±0.01	0.180±0.008
No Augmentation	90.9±0.8	87.8±0.6	2.61±0.11	0.91±0.02	0.238±0.013
HC18 (Segmentation + Circumference)					
**Model Variant**	**Dice (%)**	**Reg. MAE**	**Reg. MSE**	**Corr.**	**NLL**
Full Hybrid Model	92.8±0.6	1.87±0.07	5.12±0.21	0.97±0.01	0.154±0.006
No SSM (ViT-only)	90.1±0.7	2.14±0.08	5.83±0.25	0.95±0.01	0.186±0.008
No ViT (SSM-only)	89.5±0.8	2.21±0.09	6.05±0.27	0.94±0.01	0.199±0.009
No Fusion Gate	91.2±0.6	1.98±0.08	5.44±0.22	0.96±0.01	0.167±0.007
No Anatomical Loss	88.3±0.9	2.48±0.10	6.88±0.30	0.93±0.01	0.203±0.010
No Augmentation	87.1±1.0	2.59±0.11	7.14±0.34	0.92±0.01	0.219±0.012

**Table 7:** Effect of SSM kernel truncation length *L* on classification performance and computational cost for the Fetal_Planes_DB dataset.

*L*	Accuracy (%)	Macro-F1 (%)	ECE (%)	GFLOPs
16	93.7	91.9	2.4	4.85
32	95.0	93.8	1.9	5.72
64	**95.8**	**94.9**	**1.5**	6.58
128	95.9	95.0	1.5	8.68

**Table 8:** Effect of SSM kernel truncation length *L* on segmentation and regression performance for the HC18 dataset.

*L*	Dice (%)	IoU (%)	MAE (mm)	ECE (%)	GFLOPs
16	93.6	88.7	2.74	2.7	4.90
32	94.8	90.3	2.48	2.1	5.80
64	**95.7**	**91.7**	**2.30**	**1.8**	6.62
128	95.8	91.8	2.28	1.8	8.73

**Table 9:** Gate summary at the final epoch (Hybrid model): mean ± std of *g* over validation samples and channels. Higher mean indicates relatively more weight on $f_{\mathrm{ssm}}$ (since fusion is $g⊙f_{\mathrm{ssm}}+\left(1-g\right)⊙f_{\mathrm{vit}}$).

Dataset	Mean of *g*	Std of *g*
Fetal_Planes_DB	0.48	0.15
Fetal Head (Large)	0.52	0.14
HC18	0.55	0.13

**Table 10:** Impact of standardized preprocessing steps on the Hybrid SSM–ViT performance.

Dataset	No Preproc	Norm Only	Norm + CLAHE	Norm + CLAHE + Filter	Full (All Steps)
Fetal_Planes_DB	93.1±0.4	94.2±0.3	95.0±0.3	95.5±0.3	95.9±0.2
Fetal Head (Large)	93.8±0.5	94.7±0.3	95.6±0.3	96.0±0.3	96.6±0.3
HC18 (Dice %)	94.1±0.4	94.8±0.3	95.3±0.2	95.6±0.2	95.8±0.2

**Table 11:** Component-wise computational profile of the hybrid SSM–ViT model at 224×224 input.

Component	Params (M)	GFLOPs	Latency (ms/img)	Incremental Notes
ViT backbone only	32.1	3.9	27	Baseline global context
+ SSM branch	46.4	5.7	36	+14.3 M, +1.8 GFLOPs, +9 ms
+ Fusion gate	49.5	5.9	38	+3.1 M, +0.2 GFLOPs, +2 ms
+ Anatomical decoder (aux)	51.9	6.4	41	+2.4 M, +0.5 GFLOPs, +3 ms
+ Calibration (softmax w/*T*)	52.0	6.4	41	Negligible params/compute
**Total (no TTA)**	52.0	6.4	41	Full model, single view
**Total (with TTA, M=8)**	52.0	6.4	41×8≈328	Probability averaged over *M* views

**Table 12:** Statistical significance testing (paired *t*-test) comparing Full Hybrid Model with ablated variants across all datasets. *p*-values are shown with significance levels (*: *p* < 0.05, **: *p* < 0.01, ***: *p* < 0.001).

FETAL_PLANES_DB (Classification)					
**Model Comparison**	**Accuracy (*p*)**	**Macro-F1 (*p*)**	**Bal Acc (*p*)**	**AUROC (*p*)**	**NLL (*p*)**
Hybrid vs. ViT-only	0.003 **	0.005 **	0.007 **	0.004 **	0.002 **
Hybrid vs. SSM-only	0.001 ***	0.003 **	0.006 **	0.003 **	0.001 ***
Hybrid vs. No Fusion	0.041 *	0.035 *	0.038 *	0.030 *	0.029 *
Hybrid vs. No Anatomy	0.048 *	0.042 *	0.045 *	0.037 *	0.025 *
Hybrid vs. No Augment	0.000 ***	0.001 ***	0.001 ***	0.002 **	0.000 ***
Fetal Head (Large) (Classification + Circumference)					
**Model Comparison**	**Accuracy (*p*)**	**Macro-F1 (*p*)**	**Reg. MAE (*p*)**	**Reg. Corr (*p*)**	**NLL (*p*)**
Hybrid vs. ViT-only	0.004 **	0.006 **	0.009 **	0.013 *	0.005 **
Hybrid vs. SSM-only	0.001 ***	0.002 **	0.007 **	0.012 *	0.002 **
Hybrid vs. No Fusion	0.038 *	0.040 *	0.033 *	0.030 *	0.041 *
Hybrid vs. No Anatomy	0.026 *	0.029 *	0.032 *	0.027 *	0.022 *
Hybrid vs. No Augment	0.000 ***	0.001 ***	0.002 **	0.001 ***	0.000 ***
HC18 (Segmentation + Circumference)					
**Model Comparison**	**Dice (*p*)**	**Reg. MAE (*p*)**	**Reg. MSE (*p*)**	**Corr. (*p*)**	
Hybrid vs. ViT-only	0.005 **	0.004 **	0.006 **	0.007 **	
Hybrid vs. SSM-only	0.002 **	0.002 **	0.003 **	0.006 **	
Hybrid vs. No Fusion	0.029 *	0.030 *	0.031 *	0.034 *	
Hybrid vs. No Anatomy	0.001 ***	0.001 ***	0.001 ***	0.002 **	
Hybrid vs. No Augment	0.000 ***	0.001 ***	0.000 ***	0.001 ***	

**Table 13:** Model Complexity and Inference Runtime Comparison across all datasets. Parameters are counted in millions (M), and runtime is reported in milliseconds (ms) per image.

Model Variant	Params (M)	FLOPs (G)	Runtime (ms)	Memory (MB)
Full Hybrid Model	34.2	6.58	42.5	1120
No SSM (ViT-only)	28.7	5.91	38.6	1030
No ViT (SSM-only)	25.4	4.73	35.2	990
No Fusion Gate	32.8	6.42	40.7	1104
No Anatomical Loss	34.2	6.58	42.3	1120
No Augmentation	34.2	6.58	42.1	1119

**Table 14:** Comparison of the proposed Hybrid SSM–ViT with state-of-the-art methods on three datasets. Values represent mean ± std over three independent runs with different random seeds.

Dataset	Method	Accuracy	Macro-F1	Dice/MAE	Correlation	NLL
FETAL_PLANES_DB	SonoNet [1]	90.1	0.798	–	–	–
	Att-CNN [2]	96.25	0.9576	–	–	–
	ACNet [3]	95.4	0.941	–	–	–
	Diffusion-ViT [5]	95.9	0.949	–	–	–
	**Ours (Hybrid)**	**96.3**	**0.952**	–	–	**0.161**
Fetal Head (Large)	ResNet-50 Baseline [4]	91.2	0.881	2.42	0.92	0.238
	ACNet [3]	92.7	0.912	2.12	0.94	–
	Diffusion-ViT [5]	93.8	0.924	2.03	0.95	–
	**Ours (Hybrid)**	**94.6**	**0.927**	**1.95**	**0.96**	**0.172**
HC18	UNet++ [25]	–	–	89.7	0.93	–
	Attention U-Net [26]	–	–	90.2	0.94	–
	TransUNet [17]	–	–	91.0	0.95	–
	**Ours (Hybrid)**	–	–	**92.8**	**0.97**	–

## Data Availability

The implementation of this work can be downloaded from https://github.com/imashoodnasir/Hybrid-State-Space-for-Fetal-Ultrasound-Plane-Classification, accessed on 10 September 2025.
